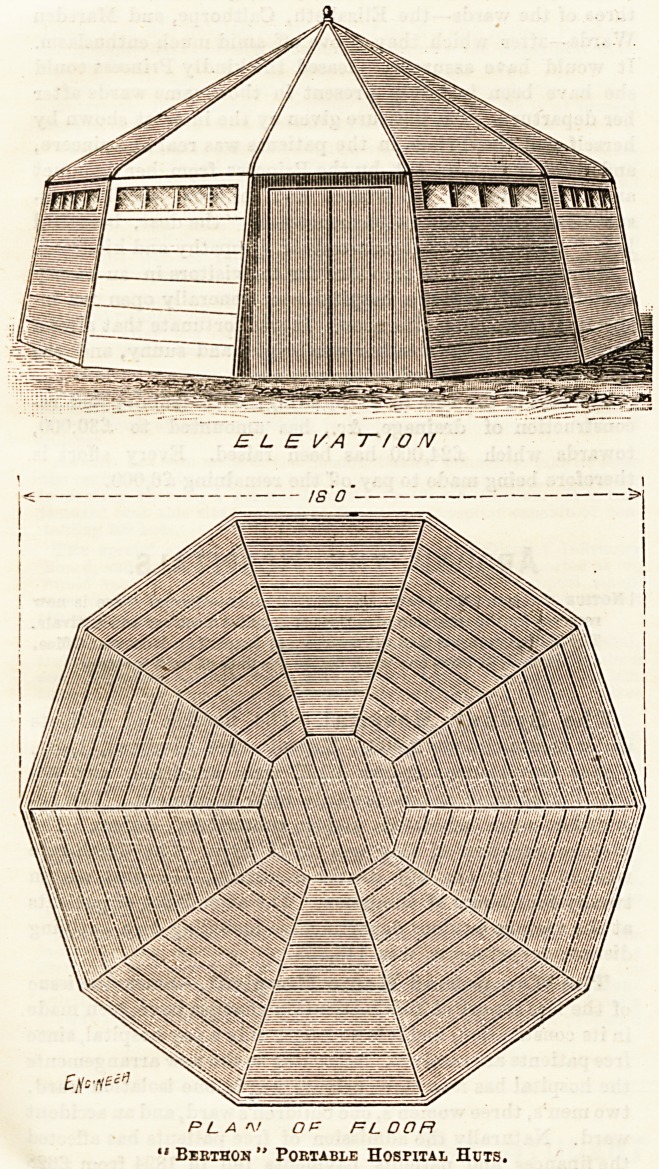# Practical Departments

**Published:** 1895-07-27

**Authors:** 


### PRACTICAL DEPARTMENTS.

### "BERTHON" PORTABLE HOSPITALS.

Quite recently we gave in these columns a description of a hospital on wheels for isolation use in rural districts. To-day we have another kind of portable isolation hospital, similar in the ends it serves, of effecting prompt and efficient isolation at small expense. These very convenient little "hospital huts" of which the accompanying illustration gives a good idea, were on view at the late Agricultural Show at Bournemouth, and enquiry elicited the fact that several were actually in use at the Sanitary Hospital at Boscombe, where they had met with the unqualified approval of Dr. Nunn, Medical Officer of Health for the district.

Dr. Nunn was good enough to give an opportunity for inspection, and all necessary information to the writer, who was much impressed by the comfortable aspect of the little huts, which were unoccupied at the time, but gave ample evidence of their sanitary excellence combined with every arrangement for the patient's comfort.

The huts are the invention of the Rev. E. L. Berthon, whose name is connected with the well-known "Berthon" boats. The roof is made on the same principle, "with timbers radiating from the apex, from which extend two plies of canvas made waterproof with flexible paint." The walls consist of two thicknesses of matched board with glass windows in each segment, and the ventilation is as good as could be wished; the fresh air entering between the double sides, and passing upwards between the two skins of the roof, escapes by ventilators at the apex. The windows also supply thorough cross-ventilation, and there is a good air space beneath the floor, so that it will be seen that the fresh air system is quite complete. Our illustration, given by permission of the inventor, shows the smaller of these two sizes in which the huts are usually made; it is ten-sided, measuring 18 by 18 feet. There are eight windows and two doors. The larger sized hut consists of the two halves with the addition of 14 feet, in three bays, between them, the length being 32 feet. The woodwork can be made fire-proof by means of asbestos paint, and it is otherwise rendered particularly safe from fire as the soap with which the canvas is saturated makes ignition difficult, and in any case the hut would only smoulder. The floor is made of segments of board, stained and varnished.

The use at Bournemouth are not in regular use, but are kept by way of precaution in case of an outbreak exhausting the accommodation within the hospital walls, and Dr. Nunn finds that the patients like their sojourn in the huts as well or better than being in the wards. They will comfortably hold three beds or four children's cots. They have been tried in all weathers and under many different conditions; they are cool in summer, owing to the double painted canvas lining to the roof, and in cold and wintry seasons it is possible and easy to keep up an even and cosy temperature with a No. 1 slow combustion (Tortoise) stove, placed in the centre, with the pipe projecting through the apex of the roof, nowhere touching wood or canvas. The huts have been proved to be thoroughly weather-tight, and indeed, in a great storm of recent years, when the roof was actually blown off one of the Sanitary Hospital's pavilions, the squat little isolation huts remained absolutely unharmed, the wind appearing almost to cause them to stick tighter to mother earth, the slightly-sloping sides of course helping largely to make and keep them steady.

As a proof of how well adapted they are for use in cold weather, we should not omit to mention that the members of the Jackson-Harmsworth Polar Expedition have lived in these huts, provided with double windows, threefold walls, and a very large stove, through the past winter in Franz Josef Land, latitude 83° 30′ N. For hospital purposes, where permanently required, Mr. Berthon informs us that they are

"BERTHON" PORTABLE HOSPITAL HUTS.

often supplied in threes, with a small covered way between, the centre one for the nurse, and one on each side for men and women respectively, with earth closets fitted up between, thus making an entirely self-contained little hospital.

Two other important details remain. First, that the price is very moderate, that of the smaller ten-sided hut being (complete) £50, and the larger, which is, as we have said, 32 by 18 feet, £65. This brings them within the reach of all sanitary authorities, and, being of so simple a construction, repairs are easy, and disinfection also. The second point is their easy portability. Many contrivances, so called "portable," really in actual practice involve a large amount of trouble, and even expense, in moving. Not so the "Berthon" hospitals, which can, the matron of the Sanitary Hospital assured us, be easily taken to pieces and removed on a wheel-barrow. There can be no doubt that they can very advisedly be used as most efficient supplements to the ordinary fever accommodation in many cases, and may be found of much value in small country districts as a substitute for a more permanent building, their reasonable cost being a recommendation not to be overlooked. It must not be imagined that we are advocating their adoption at all *instead* of a properly maintained infectious hospital, but as an adjunct, and in poor villages where removal to the nearest available hospital entails a long journey, and where there are not funds for building, they may well be substituted, and will be found to answer every necessary requirement.

### A NEW AMBULANCE.

The advantages of cycling are many, and the latest idea for turning them to account in the way of a cycle ambulance, which comes to us from Berlin, the invention of Dr. Hőnig of that city, seems to have some practical recommendations. The conveyance in question was inspected recently by a number of surgeons and ambulance and fire brigade authorities, and it would certainly seem that, as the inventor suggests, in many a small town where the expense of keeping an efficient horse ambulance would be a consideration, a carriage on the lines he proposes might be a really substantial boon. The idea is that the ambulance carriage or litter, which is covered with a detachable roof and provided with little windows, shall rest upon a frame propelled by five wheels, three in front, tricycle fashion, and two behind, the drivers thus being one at each end. They can be communicated with by the patient inside by means of a pneumatic bell.

Opinion in Berlin seemed to incline to the notion that few medical men would be sufficiently good cyclists to render the plan a success, but as with every year cycling becomes more and more popular, and the facilities for learning to ride greater, this would be a difficulty scarcely worth consideration. Moreover, the art of cycling is not so hard of acquirement but that it might be easily made a condition that surgeons officially attached to the ambulance service must master its difficulties, and there would, of course, be a thoroughly competent attendant always available. On the whole we cannot help thinking the "cycle ambulance" is worth more than a passing consideration at the hands of those charged with the management of the ambulance service in small towns.

## Figures and Tables

**Figure f1:**